# Acute exhaustive aerobic exercise training impair cardiomyocyte function and calcium handling in Sprague-Dawley rats

**DOI:** 10.1371/journal.pone.0173449

**Published:** 2017-03-08

**Authors:** Kristine Ljones, Henning Ofstad Ness, Karin Solvang-Garten, Svein Erik Gaustad, Morten Andre Høydal

**Affiliations:** 1 Department of Circulation and Medical Imaging, Norwegian University of Technology and Science (NTNU), Trondheim, Norway; 2 K. G. Jebsen Centre of Exercise in Medicine, Trondheim, Norway; Universidad de Buenos Aires, ARGENTINA

## Abstract

**Introduction:**

Recent data from long-distance endurance participants suggest that cardiac function is impaired after completion. Existing data further indicate that right ventricular function is more affected than left ventricular function. The cellular mechanisms underpinning cardiac deterioration are limited and therefore the aim of this study was to examine cardiomyocyte and molecular responses of the right and left ventricle to an acute bout of exhaustive endurance exercise.

**Materials and methods:**

Male Sprague-Dawley rats were assigned to sedentary controls or acute exhaustive endurance exercise consisting of a 120 minutes long forced treadmill run. The contractile function and Ca^2+^ handling properties in isolated cardiomyocytes, protein expression levels of sarcoplasmic reticulum Ca^2+^-ATPase and phospholamban including two of its phosphorylated states (serine 16 and threonine 17), and the mitochondrial respiration in permeabilized cardiac muscle fibers were analyzed.

**Results:**

The exercise group showed a significant reduction in cardiomyocyte fractional shortening (right ventricle 1 Hz and 3 Hz p<0.001; left ventricle 1 Hz p<0.05), intracellular Ca^2+^ amplitude (right ventricle 1 and 3 Hz p<0.001; left ventricle 1 Hz p<0.01 and 3 Hz p<0.05) and rate of diastolic Ca^2+^ decay (right ventricle 1 Hz p<0.001 and 3 Hz p<0.01; left ventricle 1 and 3 Hz p<0.01). Cardiomyocyte relaxation during diastole was only significantly prolonged at 3 Hz in the right ventricle (p<0.05) compared to sedentary controls. We found an increase in phosphorylation of phospholamban at serine 16 and threonine 17 in the left (p<0.05), but not the right, ventricle from exhaustively exercised animals. The protein expression levels of sarcoplasmic reticulum Ca^2+^-ATPase and phospholamban was not changed. Furthermore, we found a reduction in maximal oxidative phosphorylation and electron transport system capacities of mitochondrial respiration in the right (p<0.01 and p<0.05, respectively), but not the left ventricle from rats subjected to acute exhaustive treadmill exercise.

**Conclusion:**

Acute exhaustive treadmill exercise is associated with impairment of cardiomyocyte Ca^2+^ handling and mitochondrial respiration that causes depression in both contraction and diastolic relaxation of cardiomyocytes.

## Introduction

There is compelling evidence for numerous beneficial effects of regular physical activity on public health [1, 2]. A number of studies have documented the importance of regular physical activity in the prevention, risk factor modification, treatment and rehabilitation of numerous cardiovascular diseases [3–6]. However, recent data show that very strenuous aerobic exercise, e.g. attending (ultra)long-distance endurance competitions, imposes a high degree of stress on all myocardial structures. Moreover, accumulating evidence suggests that acute bouts of prolonged strenuous endurance exercise may lead to a transient cardiac dysfunction [7–15]. Many of these studies have suggested a transient impairment in systolic and/or diastolic cardiac function, and several have found a concurrent elevation in biomarkers of myocardial injury, including cardiac troponin T and/or troponin I [7–15]. The clinical significance of these changes is, however, controversial [16–20].

The right and left ventricle of the human heart originates from two separate sources during embryological development [21], and the genetic make-up and post-birth changes of the right ventricle (RV) are unique [22]. After birth the RV muscle mass is relatively reduced to approximately one-sixth of that of the left ventricle (LV) [23]. During exercise the combination of increased left atrial pressure and limited recruitable pulmonary vascular reserve capacity contribute to an increase in pulmonary artery pressures [22, 24, 25]. Hence, the afterload of the RV increases, inflicting a higher workload on the RV. In accordance with this, La Gerche et al. [24] demonstrated a disproportionate increase in RV load during exercise, as compared to LV load. While the LV end-systolic wall stress increased by 14% during exercise, the corresponding increase in the RV was 125% [24].

Most of the earlier studies of strenuous endurance exercise focused exclusively on LV function. Recent advances in echocardiographic and cardiac magnetic resonance (CMR) imaging have allowed a more comprehensive assessment of RV structure and function. A number of these studies have indicated that strenuous endurance exercise might affect RV function more profoundly than the LV; RV function may be affected even in situations where LV function appear to be preserved [10–15].

In a study of 40 athletes participating in various endurance races with a duration of 3 to 11 hours, La Gerche et al. [13] found a transient (recovery within 1 week) increase in RV volumes and a decline in RV function immediately after race completion. In contrast, LV volumes were reduced and LV function was preserved in the post-competition setting.

A meta-analysis of the acute effects of prolonged endurance exercise revealed a significant impairment in RV function while LV function appeared relatively unaffected [15]. Furthermore, in a recent study Claessen et al. [14] performed real-time CMR imaging examination of 14 male endurance athletes during incremental exercise prior to and immediately after completion of a 150-km cycling event. They reported RV dilatation and decreased RV function immediately after the race, and these alterations were even more evident during near-maximal exercise in the post-race setting. In contrast, measures of LV volume and function were unaffected. They concluded that it appeared to be an exercise-induced RV contractile impairment and that the RV, not the LV, was predominantly affected by intense endurance exercise [14].

Taken together, these data suggest that the RV is less able to sustain the intense workload during prolonged exercise. The exact cellular mechanisms responsible for RV dysfunction remains, however, unknown. The main purpose of this study was therefore to examine how an acute bout of exhaustive endurance exercise affect cardiomyocyte contractile function, Ca^2+^ handling and mitochondrial respiration in RV and LV in healthy rats. We found that acute exhaustive aerobic exercise caused depressed calcium handling and cardiomyocyte dysfunction.

## Material and methods

### Animals

Male Sprague-Dawley rats were obtained from Taconic Biosciences, Denmark. The animals were housed in individually ventilated cages, controlled at a 12:12 h dark-light cycle with a temperature of 19–22°C and humidity of 50–60% at the Comparative Medicine Core Facility, Norwegian University of Science and Technology (NTNU). They were acclimatized to the facility prior to initiation of the experimental procedures and were provided a standard pelleted rat chow and water *ad libitum*.

All experimental protocols were approved by the Norwegian Animal Research Authority (Permit Number: 4450 and 7792) and conformed to the European Directive on the Protection of Animals used for Scientific Purposes (Directive 2010/63/EU, European Parliament and Council of Europe, 2010). All surgery was performed under isoflurane anesthesia, and all efforts were made to minimize suffering.

A total of thirty animals (body weight 415 ± 42 g) were randomly assigned into four different groups. Group I (n = 5) served as sedentary controls for the analysis of contractility and Ca^2+^ handling in isolated cardiomyocytes from both RV and LV. Group II (n = 5) was subjected to an acute bout of exhaustive exercise before analysis of contractility and Ca^2+^ handling from both RV and LV. Group III (n = 10) served as sedentary controls for the analysis of mitochondrial respiration in permeabilized cardiac fibers and western blot analysis of cardiac tissue homogenate from RV and LV. Finally, group IV (n = 10) was assigned to the same exhaustive exercise protocol as group II before analysis of mitochondrial respiration and western blot analysis.

### Exercise protocol

Animals were kept sedentary until the day of the experimental procedures. Rats assigned to an acute bout of exhaustive treadmill running (group II and IV) were trained individually on a motor-driven treadmill at an inclination of 25° for a total of 120 minutes. The treadmill run started off at a pace of 6 m/min for 15 minutes. Thereafter the speed was increased with 1 m/min every 15 minute until the speed of 12 m/min was reached after 90 minutes. Finally, the pace was kept constant at 12 m/min for the last 30 minutes or until exhaustion was reached and the rat was unable to continue the session.

The treadmill was equipped with an electrical grid, giving electrical pulses of 0.2 mA, at the rear end of the treadmill belt. The electrical pulse cause discomfort but little pain, and enable accurate control of the exercise intensity. The rats were closely monitored while on the treadmill and the exercise bout was terminated if they got on to the electrical grid three consecutive times for more than 2 seconds. Based on these criteria, the treadmill run was ended early for one animal in group II (ended after 110 min) and two animals in group IV (ended after 85 min and 110 min, respectively). The body weight of the animals was reduced by 13 ± 6 g after the completion of the exercise protocol. Animals were euthanized immediately after the completion of the acute exercise bout.

### Isolation of right and left ventricular cardiomyocytes

Cardiomyocytes from exercised and control animals were isolated in order to analyze contractile and Ca^2+^ handling properties. Hearts were quickly excised from isoflurane-anesthetized and heparinized (0.3 mL 1000 IU/kg injected in the LV) animals and immersed in ice-cold modified Krebs-Henseleit bicarbonate buffer (solution A; calcium free) containing 118.5 mM NaCl, 4.7 mM KCl, 1.2 mM KH_2_PO_4_, 1.2 mM MgSO_4_*7H_2_O, 25.0 mM NaHCO_3_, 1.0 mM DL-carnitine and 11.0 mM C_6_H_12_O_6_*H_2_O, pH 7.4. Thereafter, the hearts were connected to an aortic cannula of a standard Langendorff retrograde perfusion system and cardiomyocytes were isolated as previously described by Wisløff et al. [26]. Briefly, hearts were retrogradely perfused via aorta with solution A (7.5 mL/min) equilibrated with 5% CO_2_-95% O_2_ for 5 minutes (at 37°C, pH 7.4). Followed by perfusion with solution B consisting of solution A supplemented with 295 U/mL collagenase type II (Worthington Biochemical Corporation, UK) and 0.1% bovine serum albumin, for 20 minutes (7.5 mL/min). Hearts were then cut down and placed in solution C, consisting of solution A supplemented with 1% bovine serum albumin and 1.5 mM CaCl_2_ equilibrated with 5% CO_2_-95% O_2_. The great arteries and atria were removed and the ventricles were separated into left (free wall and interventricular septum) and right ventricular tissue. The tissue was further cut into small pieces and shaken for 15 minutes (37°C, 5% CO_2_-95% O_2_, 150 rpm). The resulting cell suspensions were centrifuged for 30 seconds at 600 rpm (37°C). Fresh solution C was added to the pellet and the centrifugation step was repeated. Finally, the pellet was resuspended in solution C and filtered through a nylon mesh (250 μm).

### Contractility and Ca^2+^ handling

Isolated cardiomyocytes were loaded with Fura-2/AM for assessment of Ca^2+^ handling properties. Experiments were carried out in HEPES solution consisting of 135 mM NaCl, 5 M KCl, 1.0 mM MgCl_2_*6 H_2_O, 1.2 mM CaCl_2_, 10 mM HEPES and 8 mM glucose, pH 7. All analyzes of contractile function were performed with a standardized loading procedure, i.e. 1 μM Fura-2/AM for 10 minutes at 22°C. The cells were washed twice with fresh HEPES solution after loading. A strict loading procedure was used to avoid any difference between cells on fractional shortening by Fura-2/AM *per se*, as Fura-2/AM previously has been shown to have depressive effect on cardiomyocytes [27].

During the experiments, cells were continuously superfused with HEPES solution at 37°C. Cardiomyocytes were stimulated by bipolar electrical pulses with increasing frequencies (1–3 Hz) on an inverted epifluorescence microscope (Nikon TE-2000E, Tokyo, Japan). Cardiomyocyte function was recorded by video-based myocyte sarcomere spacing (SarcLen^™^, IonOptix, Milton, MA) and intracellular Ca^2+^ concentration was measured by detection of Fura-2/AM fluorescence by a photomultiplier tube (Optoscan, Cairn Research, Kent, UK). Upon binding of Ca^2+^, Fura-2/AM exhibits an absorption shift that is measured by excitation at 340 and 380 nm, with an emission wavelength of 510 nm. When excited at 340 nm, fluorescence emission increases with increasing intracellular Ca^2+^ concentration. At the same time, the fluorescence emission decreases with increasing intracellular Ca^2+^ concentration when excited at 380 nm. Thus, the use of Fura-2/AM minimizes the experimental variations between cells of e.g. loading, photo bleaching, varying room lightning, leakage, and compartmentations of Fura-2/AM in the cell [28].

### Protein extraction

Frozen ventricular tissue from RV and LV of sedentary controls (n = 9) and exhaustively exercised (n = 9) rats was mechanically homogenized in Pierce RIPA buffer (ThermoFisher Scientific, Waltham, MA, USA) supplemented with phosphatase inhibitor cocktail 2 and 3 (Sigma-Aldrich Co, St.Luis, MO, USA) and Complete protease inhibitor cocktail (Roche Diagnostics GmbH, Rockford, IL, USA) using a Precellys24 homogenizer (Bertin Technologies, Montigny-le-Bretonneux, France); 15 seconds at 6000 rpm followed by 15 minutes of shaking on ice. Homogenization and shaking was repeated once. The liquid was spun down at 16,000 rcf for 10 minutes, 4°C. Protein concentration of each sample was quantified spectrophotometrically according to the protocol of the kit manufacturer (Pierce BCA Assay kit, ThermoFischer Scientific, Waltham, MA, USA).

### Western blot

Samples were prepared with LDS protein loading buffer and 0.2 M 1,4-dithiothreitol and heated for 10 min at 70°C. Equal amounts of protein were loaded onto each well of NuPage Novex Bis-Tris 10% gels (Life Technologies, Carlsbad, CA, USA). Chameleon Duo Pre-Stained Protein Ladder (LI-COR Biosciences, Lincoln, NE, USA) was used as a standard. Gels were run in BOLT MES Running buffer (Life Technologies, Carlsbad, CA, USA) for 80 minutes at 150 V, 4°C. Proteins were then transferred onto a methanol activated Immobilon-FL PVDF membranes (Merck Millipore Ltd., Carrigtwohill, Ireland) at 20 V, 90 minutes, 4°C, in BOLT Transfer buffer (Life Technologies, Carlsbad, CA, USA). After the transfer, membranes were washed, dehydrated and cut according to the protein ladder to separate SERCA2a, β-tubulin and phospholamban with its phosphorylated states (serine 16 and threonine 17).

Membranes were rehydrated and incubated in Odyssey TBS Blocking buffer (LI-COR Biosciences, Lincoln, NE, USA) for 1 hour at room temperature prior to incubation with primary antibodies diluted in Odyssey TBS Blocking buffer supplemented with 0.2% Tween over night at 4°C, constant shaking. Primary antibodies used were commercially available and included polyclonal rabbit anti-SERCA2a (#A010-20) 1:10,000, monoclonal mouse anti-phospholamban (#A010-14) 1:5,000, polyclonal rabbit anti-serine 16 phosphorylated phospholamban (pPLB Ser16; #A010-12) 1:2,500, and polyclonal rabbit anti-threonine 17 phosphorylated phospholamban (pPLB Thr17; #A010-13) 1:2,500, all from Badrilla Ltd. (Leeds, UK). Polyclonal anti-β-tubulin (#AB6046, Abcam, Cambridge, UK) 1:10,000 was used to measure protein expression levels of β-tubulin as a loading control.

Membranes were washed in TBS Tween buffer before incubation with fluorescent dye-conjugated secondary antibodies for 1 hour at room temperature, constant shaking. Secondary antibodies: Donkey anti-rabbit IRDye 680LT 1:40,000 and goat anti-mouse IRDye 800CW 1:15,000 (both from LI-COR Biosciences, Lincoln, NE, USA), diluted in Odyssey TBS Blocking buffer supplemented with 0.2% Tween and 0.01% SDS. Membranes were washed in TBS Tween, followed by TBS, air dried and scanned using the Odyssey FC Imaging system (LI-COR Biosciences, Lincoln, NE, USA). Protein bands were analyzed using Image Studio Ver3.1 (LI-COR Biosciences, Lincoln, NE, USA). For PLB, pPLB Ser16 and pPLB Thr17 the monomer band was quantified. Band signals were corrected for local background and SERCA, PLB, pPLB Ser16 and pPLB Thr17 signals were normalized to β-tubulin and a common sample that was run on all gels. Samples were analyzed with four technical replicates. Normalized signals were converted to fold change for exhaustively exercised rats compared to sedentary controls.

### Preparation of permeabilized cardiac fiber bundles

Saponin-permeabilized cardiac fiber bundles from RV and LV were prepared from exhaustively exercised and sedentary control animals in order to assess mitochondrial respiration rates. Hearts were quickly excised from isoflurane-anesthetized animals and small pieces of RV and LV apical free wall were cut off and kept in ice-cold biopsy preservation solution (BIOPS) consisting of 2.77 mM CaK_2_-ethylene glycol tetraacetic acid (CaK_2_EGTA), 7.23 mM K_2_EGTA, 5.77 mM Na_2_ATP, 6.56 mM MgCl_2_*6 H_2_O, 20 mM taurine, 15 mM Na_2_phosphocreatine, 20 mM imidazole, 0.5 mM dithiothretiol, and 50 mM K-MES hydrate, adjusted to pH 7.1 at 0°C [29].

Permeabilization of cardiac muscle fibers for high-resolution respirometry was achieved by a combination of mechanical and chemical permeabilization as previously described [29, 30]. Briefly, excised cardiac tissue was kept in ice-cold BIOPS, connective tissue was trimmed off, the tissue was divided into subsamples, and each subsample was dissected into a meshwork of fibers. Complete permeabilization of the plasma membrane was achieved by gentle agitation in BIOPS supplemented with 50 mg/mL saponin for 20 minutes at 4°C. Fiber bundles were then rinsed by gentle agitation for 10 minutes at 4°C in mitochondrial respiration medium (MiR05) consisting of 0.5 mM EGTA, 3 mM MgCl_2_*6 H_2_O, 60 mM K-lactobionate, 20 mM taurine, 10 mM KH_2_PO_4_, 20 mM HEPES, 110 mM sucrose and 1 g/L bovine serum albumin (essentially fatty acid free), pH adjusted to 7.1 at 30°C [29].

### High-resolution respirometry

Measurements of respiratory flux in permeabilized cardiac fiber bundles were performed using the high-resolution respiratory system Oxygraph-2k (OROBOROS, Innsbruck, Austria). Experiments were carried out with samples of 0.7 to 1.1 mg wet weight in 2 mL continuously stirred MiR05, at 37°C and hyperoxygenated levels to prevent limitation of oxygen diffusion [29]. DatLab software (OROBOROS INSTRUMENTS, Innsbruck, Austria) was used for data acquisition and analysis. For each heart, samples from the LV and RV was analyzed in triplicates.

Characterization of the mitochondrial respiratory complexes was obtained using the following sequential substrate-uncoupler-inhibitor titration (SUIT) protocol adapted from a previous description by Lemieux et al. [31] (final concentrations): 10 mM glutamate, 2 mM malate and 5 mM pyruvate, 5 mM ADP, 10 μM cytochrome c, 10 mM succinate, 0.5 μM steps of carbonyl cyanide m-chloro phenyl hydrazone (CCCP; titration up to optimum concentration of 0.5–2.0 μM), 0.5 μM rotenone, 5 mM malonic acid and 2.5 μM antimycin A, 2 mM ascorbate and 0.5 mM *N*,*N*,*N’*,*N’*-tetra-methyl-*p*-phenylenediamine (TMPD) and finally, ≥ 50 mM sodium azide.

Respiratory fluxes were corrected online for instrumental background determined at experimental oxygen levels [32], and weight-specific oxygen flux (pmol O_2_ × s^-1^ × mg^-1^ wet weight) was calculated using the DatLab software. In addition, residual oxygen consumption (ROX) was subtracted from respiratory fluxes of complex I and II in the different respiratory states. Complex IV respiration (ascorbate + TMPD) was corrected by subtracting background oxygen flux measured in the presence of azide. Oxidative phosphorylation (OXPHOS) control ratio was calculated as the ratio between OXPHOS capacity and electron transport system (ETS) capacity with combined complex I and II substrates. LEAK/OXPHOS coupling control ratio (inverse respiratory control ratio (RCR)) was calculated as the ratio between LEAK respiration with complex I substrate and OXPHOS capacity with combined complex I and II substrates.

### Statistics

Statistics were performed using IBM SPSS Statistics 21 (IBM Corporation, Armonk, NY). All data were tested with Shapiro-Wilk test of normality. Measures of contractility, Ca^2+^ handling and mitochondrial respiration of RV and LV were compared between exhaustively exercised animals and control animals using Levene’s test of equality of variances followed by independent Student’s *t*-tests. Measurements of protein expression levels of SERCA2a, PLB, pPLB Ser16 and pPLB Thr17 in RV and LV were compared between exhaustively exercised and sedentary control animals. Fold change data were Log2 transformed prior to statistical testing with independent samples Kruskal-Wallis test. P ≤ 0.05 was considered statistically significant. Data are presented as means ± SD.

## Results

The main findings of the present study were impaired fractional shortening and altered systolic and diastolic Ca^2+^ handling in isolated cardiomyocytes from both RV and LV of animals subjected to acute exhaustive aerobic exercise, compared to sedentary controls. The protein expression of pPLB Ser16 and pPLB Thr17 was increased in LV, but not RV, of exhaustively exercised animals compared to sedentary controls. In addition, impaired cardiomyocyte relengthening and altered mitochondrial respiration in cardiac muscle fibers from RV, but not LV, of animals subjected to acute exhaustive treadmill exercise, compared to sedentary controls.

### Cardiomyocyte contractility

**Fig 1:**
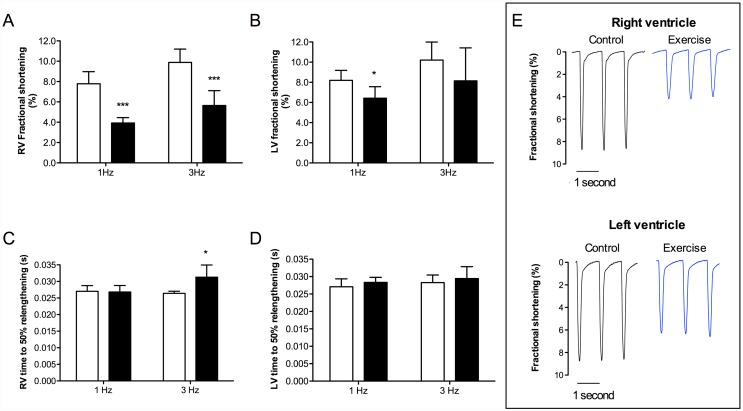
Fractional shortening and relengthening of isolated cardiomyocytes from rats subjected to acute exhaustive treadmill exercise (black bars) compared to sedentary controls (white bars). Electrical stimulation frequencies 1 and 3 Hz, n = 5. (a) Fractional shortening in RV; (b) fractional shortening in LV; (c) time to 50% relengthening in RV; (d) time to 50% relengthening in LV; and (e) representative recordings of fractional shortening in RV and LV (stimulated at 1 Hz), from sedentary and exhaustively exercised rats. * p < 0.05, *** p < 0.001.

Fractional shortening was depressed in both RV (Fig 1a; 1 Hz and 3 Hz p < 0.001) and LV (Fig 1b; 1 Hz p < 0.05) from exhausted animals. In regard to diastolic relaxation, time to 50% relengthening was significantly prolonged in RV from exhausted animals when stimulated at 3 Hz (Fig 1c; p < 0.05). In contrast, there were no significant differences in time to 50% relengthening in LV from exhausted animals at any of the stimulation frequencies (Fig 1d).

### Ca^2+^ handling

**Fig 2:**
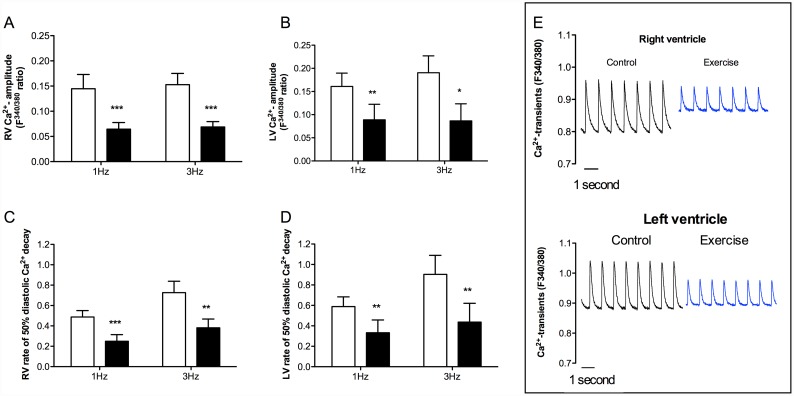
Ca^2+^ handling in isolated cardiomyocytes from RV and LV of animals subjected to acute exhaustive treadmill exercise (black bars) compared to sedentary controls (white bars). Stimulation frequencies 1 and 3 Hz, n = 5. (a) Ca^2+^ transient amplitude in RV; (b) Ca^2+^ transient amplitude in LV; (c) rate of 50% diastolic Ca^2+^ decay in RV; (d) rate of 50% diastolic Ca^2+^ decay in LV; and (e) representative recordings of Ca^2+^ transients in cardiomyocytes from RV and LV (stimulated at 1 Hz). * p < 0.05, ** p < 0.01, *** p < 0.001.

Ca^2+^ transient amplitude in both RV and LV was significantly reduced in exhausted animals (Fig 2a and 2b; RV 1 and 3 Hz p < 0.001, LV 1 Hz p < 0.01 and 3 Hz p < 0.05). In parallel, the removal rate of Ca^2+^ from cytosol during cardiomyocyte relaxation (time from peak amplitude to 50% decay of the diastolic Ca^2+^ transient), was significantly impaired in both RV and LV in exhausted animals (Fig 2c and 2d; RV 1 Hz p < 0.001 and 3 Hz p < 0.01, LV 1 and 3 Hz p < 0.01). This increased time for Ca^2+^ removal also caused a tendency of an upward shift of diastolic Ca^2+^ levels in the RV of trained animals, which is displayed in exemplary recordings (p = 0.07, Fig 2c).

### Protein expression levels

**Fig 3:**
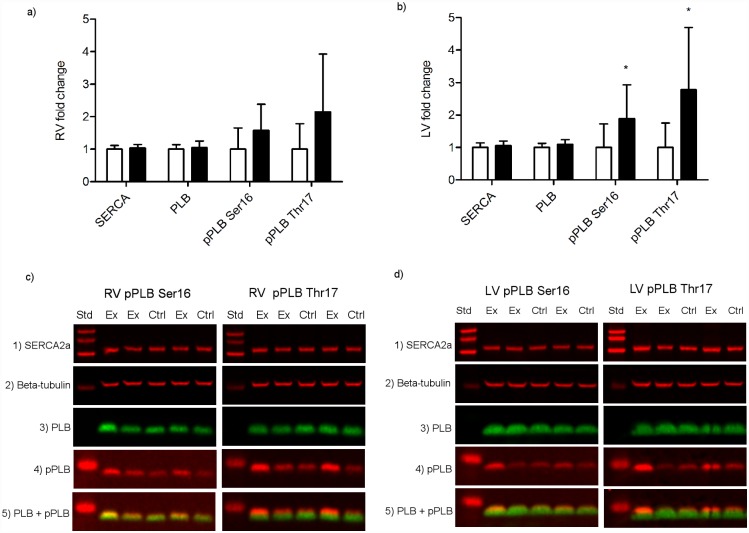
Protein expression levels in homogenized cardiac tissue from RV and LV of rats subjected to acute exhaustive treadmill exercise compared to sedentary controls, n = 9. (a, b) Protein expression levels in (a) RV and (b) LV from sedentary controls (white bars) and exhaustively exercised rats (black bars); (c, d) Exemplary western blots of protein expression levels of SERCA2a (panel 1), β-tubulin (panel 2), PLB (panel 3) and (c) left pPLB Ser16 (panel 4) and combined PLB (green) and pPLB Ser16 (red) (panel 5), right pPLB Thr17 (panel 4) and combined PLB (green) and pPLB Thr17 (red) (panel 5) in RV, (d) left pPLB Ser16 (panel 4) and combined PLB (green) and pPLB Ser16 (red) (panel 5), and right pPLB Thr17 (panel 4) and combined PLB (green) and pPLB Thr17 (red) (panel 5) in LV. SERCA2a, sarcoplasmic reticulum Ca^2+^-ATPase 2a; PLB, phospholamban; pPLB Ser16, phospholamban phosphorylated at serine 16; pPLB Thr17, phospholamban phosphorylated at threonine 17; Ctrl, control; Ex, exhaustively exercised. * p < 0.05.

The protein expression levels of pPLB Ser16 (p = 0.047) and pPLB Thr17 (p = 0.019) was significantly increased in LV from exhaustively exercised rats compared to sedentary rats (Fig 3b). In regard to the RV, there was a trend toward increased expression levels of pPLB Thr17 in exhaustively exercised rats, however, it did not reach statistical significance (p = 0.058) (Fig 3a). The protein expression levels of SERCA2a and PLB was not significantly changed by exhaustive exercise neither in LV nor RV.

### Mitochondrial respiration

**Fig 4:**
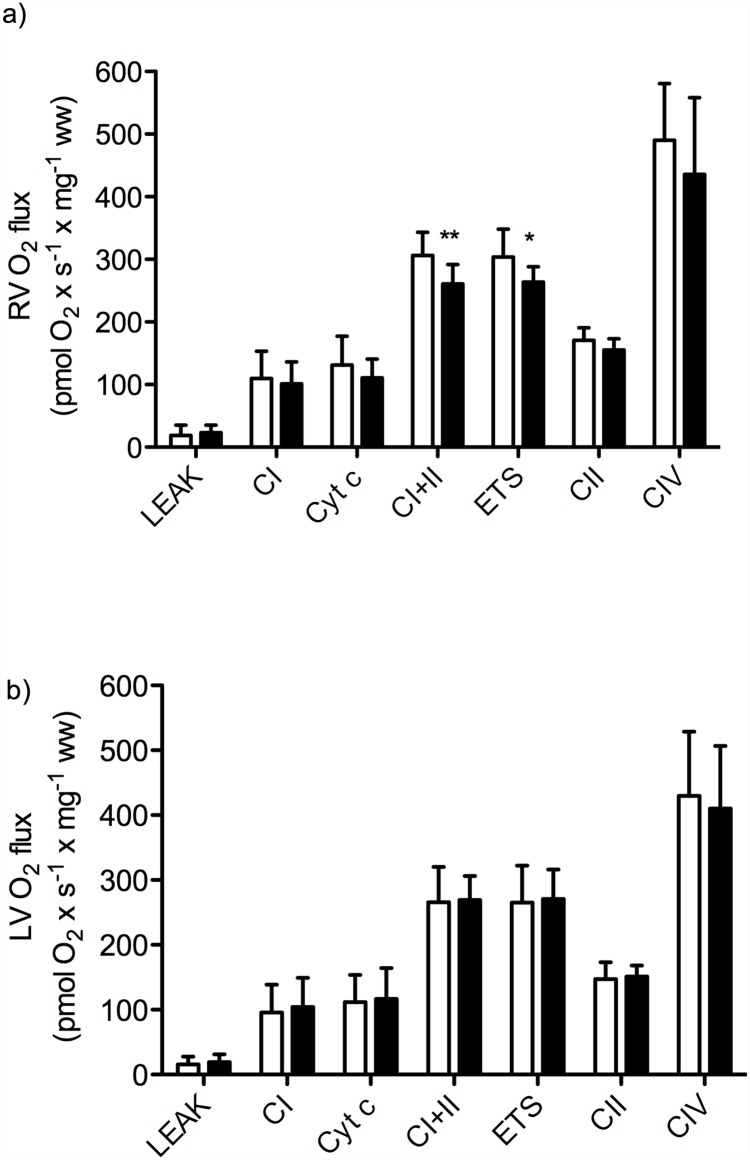
Mass-specific mitochondrial respiration in permeabilized cardiac fibers from the (a) RV and (b) LV of animals subjected to acute exhaustive treadmill exercise (black bars) compared to sedentary controls (white bars), n = 10. LEAK, LEAK respiration; CI, OXPHOS capacity with complex I substrates; Cyt c, cytochrome c; CI+II, OXPHOS capacity with combined complex I and II substrates; ETS, electron transport system capacity with combined complex I and II substrates (uncoupled respiration); CII, uncoupled respiration with complex II substrate after inhibition of complex I with rotenone; CIV, complex IV respiration; ww, wet weight. * p < 0.05, ** p < 0.01.

In RV from exhausted rats, we found significantly reduced mass-specific mitochondrial respiration for combined complex I and II substrates OXPHOS and ETS capacities (Fig 4a; OXPHOS combined complex I and II substrates p < 0.01, ETS capacity p < 0.05). In addition, there was a trend toward reduced uncoupled respiration with complex II substrate and rotenone for RV, however, this did not reach statistical significance (Fig 4a; p = 0.08). Mass-specific LEAK respiration, complex I OXPHOS capacity and complex IV respiration was not statistically different in RV from exhausted animals, compared to sedentary controls. Furthermore, there were no statistically significant differences in LV mass-specific mitochondrial respiration rates in exhausted animals compared to sedentary controls (LEAK respiration, complex I OXPHOS capacity, combined complex I and complex II OXPHOS capacity, combined complex I and complex II ETS capacity, uncoupled complex II respiration, and complex IV respiration) (Fig 4b).

In all groups, the lack of significant increase in respiration rate after the addition of cytochrome c confirmed the integrity of the outer mitochondrial membrane (Fig 4). Moreover, uncoupling of respiration with titrations of CCCP did not result in an increase in the combined complex I and II supported respiration either in RV or LV, indicating that the phosphorylation system did not exert a limiting effect on the electron transport system. Furthermore, the OXPHOS control ratio and the LEAK coupling control ratio were not significantly changed by exhaustive exercise in neither RV nor LV.

## Discussion

Our study documented a reduction in Ca^2+^ amplitude, an increase in time to 50% diastolic Ca^2+^ removal and reduced fractional shortening in both RV and LV, as well as an increase in time to 50% relengthening in RV, after acute exhaustive treadmill exercise, compared to sedentary controls. The protein expression levels of phosphorylated phospholamban (pPLB Ser16 and pPLB Thr17) was significantly increased in LV, but not RV, from exhaustively exercised animals compared to sedentary controls. The study also showed a reduction in OXPHOS and ETS capacities of mitochondrial respiration with combined complex I and II substrates in RV, but not LV, from rats subjected to acute exhaustive treadmill exercise.

### Reduced fractional shortening and impaired diastolic relaxation

The current study documented a reduction in fractional shortening of isolated cardiomyocytes from both RV and LV from rats subjected to acute exhaustive treadmill exercise. This is in agreement with the recent findings of Oláh et al. [33], that reported deterioration of LV performance, with impaired contractility and mechanoenergetics measured by LV pressure-volume recordings, in exhausted animals after 3 hours forced swim in young adult rats [33].

Regarding diastolic relaxation, we found a significant increase in time to 50% relengthening in cardiomyocytes from the RV (stimulated at 1 Hz), but not the LV, from exhausted rats. This indicates a delayed relaxation of the cardiomyocytes in the RV, but not the LV. These findings are paralleled by the previous data from Oláh et al. [33] showing no changes in diastolic LV function in exhausted animals by means of pressure-volume analyses. The data on cardiomyocyte function from the present study is also in line with recent findings of a more prominent deterioration in RV function, as compared to LV function, in humans during and immediately after prolonged endurance exercise and events [12–15, 34]. However, despite that the present data show changes in RV cardiomyocyte function to be a significant component of deteriorated cardiac function following exhaustive exercise, it is important to note that cardiac function *in vivo* is dependent on preload, afterload and the inotropic state of the myocardium, and is regulated by several different mechanisms. Thus, the observed decline in cardiac performance during and immediately after prolonged endurance exercise is likely to be more complex than solely a reduction in cardiomyocyte contractility.

### Reduced Ca^2+^ amplitude and decreased rate of Ca^2+^ decay

Concurrent with the reduction in fractional shortening and increased time to relengthening in exhausted animals, we showed a decline in Ca^2+^ transient amplitude in both RV and LV. Changes in Ca^2+^ transient amplitude or duration are among the main mechanisms by which the force of cardiac contraction is regulated. In agreement with the observed reduction in Ca^2+^ transient amplitude, Maher et al. [35] reported a depression of myocardial function in terms of force-velocity and length-active tension relations in LV trabecular muscle from rats subjected to exhaustive treadmill exercise. Furthermore, the reduced Ca^2+^ transient amplitude is a plausible explanation for the reduced fractional shortening observed in our study and the reduced cardiac contractile function previously observed after exhaustive swimming in rats by Oláh et al. [33].

In regard to diastolic parameters, we found a significant decrease in rate of 50% diastolic Ca^2+^ decay in cardiomyocytes from the RV and LV of rats subjected to acute exhaustive treadmill exercise. These data indicate a deterioration of Ca^2+^ clearance and supports the observed increase in time to 50% relengthening in RV from exhausted animals. Because SERCA2a accounts for approximately 92% of the Ca^2+^ removal during diastole in healthy rat hearts [36], an impairment in SERCA2a activity would normally be the most plausible explanation for the changes in diastolic Ca^2+^-removal observed in the present study. Moreover, reduced re-uptake of Ca^2+^ into SR would result in lower SR Ca^2+^ content and subsequent reduced SR Ca^2+^ release and Ca^2+^ amplitude. This could theoretically explain our finding of reduced Ca^2+^ transient amplitude in exhaustively exercised rats. Previous studies have, however, reported conflicting results on SERCA2a activity following acute exercise. A previous study by Pierce et al. [37] found that SR Ca^2+^ uptake was depressed immediately after an acute exhaustive swim bout in rats, and mitochondrial Ca^2^ uptake and sarcolemmal Ca^2+^ ATPases were unaffected, whereas a study by Delgado et al. [38] found that SERCA2a activity was not modified. In addition to intracellular Ca^2+^ concentration, phospholamban (PLB) is a key modulator of SERCA2a activity [39]. In its dephosphoryated state PLB act as an inhibitor of SERCA2a activity and phosphorylation of PLB at serine 16 or threonine 17 by protein kinase K (PKA) or Ca^2+^/calmodulin-dependent protein kinase II (CaMKII), respectively, has been shown to release the inhibition of SERCA2a. Accordingly, an increase in the PLB-to-SERCA2a protein expression ratio or a decrease in pPLB Ser16 and pPLB Thr17 could result in a reduction in SR Ca^2+^ uptake. It is well documented that β-adrenergic stimulation mediates phosphorylation of PLB Ser16 via activation of PKA [39]. Furthermore, a previous study by Kemi et al. [40] showed that a 6-week aerobic interval training program brought about an increase in phosphorylation of PLB at threonine 17, but not serine 16, and this increase was paralleled by an increase in phosphorylation of CaMKIIδ (the most prevalent CaMKII isoform in cardiomyocytes) at threonine 287, indicating activation [40]. Data from the present study reported no changes in the protein expression levels of SERCA2a and PLB in neither RV nor LV. Furthermore, the levels of pPLB Ser16 and pPLB Thr17 was significantly increased in LV, but not RV from exhausted rats compared to sedentary controls. Our data on impaired diastolic Ca^2+^ removal following acute exhaustive treadmill exercise are therefore not explained by changes in SERCA2a protein expression levels nor by PLB and pPLB status. The data on protein regulation from the present study would on the contrary normally cause the opposite effect with more efficient SERCA2a function and faster diastolic Ca^2+^ removal. Accordingly, other compensatory mechanisms may be present. A potential explanation could be the involvement of redox signaling that previously has been indicated in both physiological and pathophysiological cardiac processes (reviewed by Burgoyne et al. [41]). Moreover, many studies have indicated that acute exhaustive exercise may lead to oxidative and/or nitrosative stress [33, 42, 43]. Furthermore, several of the proteins involved in the excitation contraction coupling of cardiomyocytes have been shown to be influenced by oxidative modifications [44]. Among others, studies have suggested SERCA2a to be redox regulated by both direct modifications of SERCA2a itself, and indirect via regulation of PLB by oxidative modifications of its phosphoregulators PKA and CaMKII [41, 44]. Further studies are therefore needed to clarify the role of oxidative and/or nitrosative modification following exercise training.

A different and more direct explanation from the data in the present study is our findings of reduced maximal oxidative phosphorylation capacity of cardiac muscle fiber bundles from RV, but not LV, from animals subjected to acute exhaustive exercise, compared to sedentary controls. Mitochondrial dysfunction in permeabilized fibers could be the result of defects both upstream and downstream to the electron transport system, in addition to defects of the electron transport system itself. The increased cardiac workload during exhaustive endurance exercise greatly increases the energy demand of the heart, especially for the ATPases responsible for contraction, Ca^2+^ removal and relaxation. SERCA2a has been shown to be one of the main ATP consumers in cardiomyocytes [39].

Hence, a shortage in the ATP supply could affect the activity of SERCA2a and thereby explain some of the impaired clearance of Ca^2+^ observed in the present study.

Previous studies of acute effects of exhaustive endurance exercise in various animal models have demonstrated varying degrees of changes and damage to the cardiac mitochondrial ultrastructure; ranging from no detected alterations [35, 45], mitochondrial hypertrophy with preserved structural integrity [46], to swelling and more extensive disruption [47–50]. The discrepancies in findings have been suggested to be a result of a combination of different animal models, various exercise modes and diverse exercise intensities and durations. In the present work, the integrity of the outer mitochondrial membrane was tested by means of adding exogenous cytochrome c as part of the SUIT protocol used for high-resolution respirometric measurements. Furthermore, if the integrity of the inner mitochondrial membrane was disrupted one would expect an alteration in the coupling state of the oxidative phosphorylation [51]; nonetheless LEAK/OXPHOS coupling control ratio remained unaltered by the acute exhaustive treadmill run.

The SUIT protocol used in the present study assessed LEAK respiration, the respiratory pathways of complex I, II, IV and converging electrons from complex I and II, as well as the potential limitation of the electron transport system by the phosphorylation system. Our results showed a high OXPHOS control ratio for both RV and LV, and it remained unaltered by the acute exhaustive treadmill run. This indicate that the electron transport system was not limited by the phosphorylation system in sedentary controls and that the decline in maximal OXPHOS capacity after the acute exhaustive treadmill exercise could not be explained by an acquired limitation by the phosphorylation system. The results did not reveal any significant deterioration in the isolated respiratory pathways of any one of the electron transport complexes tested.

Results from previous studies of the effects of acute exhaustive exercise are contradictory; reporting unaltered [52], increased [53], or reduced [50] complex I and II-linked myocardial mitochondrial respiration rates. Terblanche et al. [52] found respiration supported by complex I or II-linked substrates to be unaltered by an acute bout of exhaustive treadmill running in homogenates of rat hearts, while the capacity of palmitoylcarnitine and malate supported respiration was significantly reduced. This is in line with the findings of our study, however, we found a trend toward reduced complex II activity in RV. Taylor et al. [50] documented a reduction in mitochondrial respiration with complex I, II and IV-linked substrates in isolated myocardial mitochondria from guinea pigs after exhaustive treadmill exercise, compared to sedentary controls. However, they noted that the ADP/O and respiratory control ratios remained unaltered, except for the respiratory control ratio with glutamate as substrate. In contrast, Ji and Mitchell [53] reported an increase in OXPHOS capacities with both complex I and II-linked substrates in isolated myocardial mitochondria from rats subjected to acute exhaustive treadmill exercise. They also reported increased LEAK respiration with all substrates analyzed. Moreover, the exhaustive treadmill protocols employed by Terblanche et al. [52], Taylor et al. [50], and Ji and Mitchell [53] deviate from the one used in our study in terms of intensity and duration. Furthermore, they did not distinguish between mitochondrial respiration of RV vs. LV, and the activity of the different respiratory pathways were tested separately and not as part of a SUIT protocol as in our study; no measures of OXPHOS capacity with converging electrons from complex I and II were assessed.

Mitochondrial respiration depends on the continuous flow of metabolites into the mitochondrial matrix. The substances used as complex I and II-linked substrates were added as intermediates of the citric acid cycle (glutamate, malate, pyruvate and succinate); depending on the dehydrogenases of the citric acid cycle to generate reduced nicotinamide adenine dinucleotide (NADH) and reduced flavin adenine dinucleotide (FADH_2_) for the electron transport system. Hence, a decline in the citric acid cycle activity would affect the activity of the electron transport system [54]. Furthermore, many of the substrates require carriers in order to cross the inner mitochondrial membrane [55]. Defects in these transmembrane carriers would induce a restriction of the respiration [54].

The exact mechanism of the observed reduction in maximal OXPHOS capacity after an acute bout of exhaustive treadmill exercise remains, therefore, elusive. It is, however, plausible that the disproportionate affection of RV function, as compared to LV function, observed after prolonged strenuous endurance exercise is linked to differing mitochondrial respiratory capacities in RV and LV, reflected by the divergent mitochondrial respiratory responses noted in the present work.

### Limitations

Isoflurane is a widely used and preferred anesthetic in animal studies as it causes less cardiodepressant effects than general injectable anesthetics [56]. However, isoflurane has been shown to be both cardiodepressant and cause a concentration-dependent reduction in complex I and III-linked mitochondrial respiration [56–58]. Isoflurane concentrations were adjusted as low as possible and the same procedure was applied for all animals. Therefore, it is unlikely that it influenced the differences observed between the groups.

In general, there are considerable limitations to *in vitro* studies. Although they afford close control of various parameters and facilitate studies of cellular and molecular mechanisms, they also limit the complex physiological interplay and signal transduction of the cells in their *in vivo* environment. Furthermore, the preparation of isolated cardiomyocytes and permeabilized cardiac muscle fibers may affect the cells and exert selective pressure. There is, however, no reason to believe that the experimental procedures have influenced the differences in various parameters observed between the groups, as all samples were treated similarly. Moreover, the experiments in the present study were performed on cardiac samples from rats subjected to acute exhaustive treadmill exercise. The animal model was chosen as human cardiac samples are scarce. As for all animal models, one should always interpret the translational value of the finding with caution. Additionally, the use of forced exercise has been shown to generate a stress response in animals that might influence on the observed responses to exhaustive exercise [59, 60].

This study includes no follow-up studies in order to determine whether the observed decline in cardiac function after an acute exhaustive treadmill run is transient or could have more persistent components and the effects of repeated exhaustive exercise bouts. Most studies of participants in long-distance endurance events have indicated that the acute effects observed are of a transient nature [7–9, 12–15]. However, several studies have suggested that repeated exposure to prolonged endurance exercise and long-distance endurance events might entail an increased risk of cardiac fibrosis and/or arrhythmias [13, 61–63].

## Conclusion

The findings of the present study show that acute exhaustive treadmill exercise is associated with a depression in the intrinsic contractile state and an impairment of the relaxation of the myocardium. Furthermore, this functional changes in the RV and LV response to exhaustive exercise appears to be associated with alterations of Ca^2+^ handling and might in part be linked to differences in mitochondrial respiratory capacities of the RV and the LV. Our findings are in conjunction with, and offer a plausible mechanism for the previously observed transient reduction in cardiac function after prolonged strenuous endurance exercise.
