# Correction: Acute exhaustive aerobic exercise training impair cardiomyocyte function and calcium handling in Sprague-Dawley rats

**DOI:** 10.1371/journal.pone.0202060

**Published:** 2018-08-03

**Authors:** The PLOS ONE Staff

The fifth author is listed incorrectly in the citation. The correct citation is: Ljones K, Ness HO, Solvang-Garten K, Gaustad SE, Høydal MA (2017) Acute exhaustive aerobic exercise training impair cardiomyocyte function and calcium handling in Sprague-Dawley rats. PLoS ONE 12(3): e0173449. https://doi.org/10.1371/journal.pone.0173449. The publisher apologizes for the error.
